# Perfectionism Profiles and Motivation to Exercise Based on Self-Determination Theory

**DOI:** 10.3390/ijerph17093206

**Published:** 2020-05-05

**Authors:** María Vicent, Ricardo Sanmartín, Oswaldo Vásconez-Rubio, José Manuel García-Fernández

**Affiliations:** 1 Department of Developmental Psychology and Teaching, Faculty of Education, University of Alicante, Apdo. Correos, 03080 San Vicente del Raspeig, Spain; ricardo.sanmartin@ua.es (R.S.); josemagf@ua.es (J.M.G.-F.); 2 Faculty of Physical Culture, Central University of Ecuador, Av. Mariscal Sucre, Quito 170129, Ecuador; jhoban77@yahoo.com

**Keywords:** perfectionism profiles, perfectionistic strivings, perfectionistic concerns, self-determination theory, exercise

## Abstract

This study complements extant variable-centered research that focus on the relationship between perfectionism and the autonomous and controlled motivation to exercise. A person-centered approach is used for identifying perfectionism profiles as well as analyzing inter-profile differences in terms of the six regulatory styles located on the autonomy-control continuum. A sample of 597 (*M*_age_ = 22.08, *SD* = 3.33) Ecuadorian undergraduates enrolled in a sport science degree program was employed. Latent Profile Analysis based on two higher-order perfectionism dimensions, Perfectionistic Strivings (PS) and Perfectionistic Concerns (PC), supported a four-class solution: *Non-Perfectionists* (low PS and PC), *Adaptive Perfectionists* (high PS and low PC)*, Maladaptive Perfectionists* (high PS and PC)*,* and *Moderate Perfectionists* (moderate PS and PC). *Adaptive Perfectionists* obtained the highest means on Intrinsic, Integrated, and Identified regulations. However, these differences where only significant when compared with *Moderate Perfectionists,* and only in the case of Integrated regulation, in comparison with *Non-Perfectionists*. In contrast, *Maladaptive Perfectionists* obtained significantly higher scores on Introjected and External regulations as well as on Amotivation than the other three classes. Results are discussed in light of Self-Determination Theory.

## 1. Introduction

There is a certain consensus in the fact that perfectionism can be considered as a multidimensional trait of personality defined by two higher-order dimensions, Perfectionistic Strivings (PS) and Perfectionistic Concerns (PC), which capture the underlying structure of instruments design to assess this construct. PS reflects the desire to reach perfection and to pursue unrealistically high standards; representing the adaptive, or at least not maladaptive, dimension of perfectionism. In contrast, PC is considered a maladaptive dimension that involves aspects associated with self-criticism, concerns over making mistakes, fears about social negative evaluation, and lack of satisfaction with achievements [1].

### 1.1. Perfectionism and Exercise 

Research on perfectionism in sport and exercise has significantly increased during the last 10 years [2]. This growing interest could be partly due to the “perfectionism paradox” [3,4]. This is the term that Flett and Hewitt [3,4] used to explain the fact that sport and exercise seem to encourage perfectionism, whereas perfectionism, in turn, might act as a vulnerability factor for athletes and exercisers. Indeed, perfectionism has been associated with a wide range of problems in the sport and exercise domain, such as burnout [5], training distress [6], favorable attitudes toward doping [7], negative affect and intentions to drop out [8], depression, worry, anxiety [9], etc. However, the consequences of perfectionism vary depending on the dimension analyzed. Hill et al. [10] performed a meta-analytical review about multidimensional perfectionism in sport and exercise, concluding that PC was clearly maladaptive, characterized by a negative pattern of motivation regulation and emotion/well-being, as well as no effects in terms of athletic performance. By contrast, PS showed an ambiguous pattern of relationships, since it was characterized by a mix of maladaptive and adaptive motivation and emotion/wellbeing, and adequate performance. Therefore, some perfectionist dimensions contain greater potential for vulnerability than others, and even those that appear to be more adaptive (such as PS) hold some perils for exercisers and athletes [2,11,12].

### 1.2. Self-Determination Theory and Exercise

Self-Determination Theory (SDT) [13] is a broad empirically based meta-theory widely employed to understand motivated behavior in the sport and exercise domains [14,15,16]. SDT considers different types of motivation located on an autonomy-control continuum between intrinsic motivation and amotivation [17]. In the specific context of exercise, intrinsically motivated behaviors are those performed because of the inherent pleasure that accompanies practicing exercise, and it represents the most autonomous regulation. On the contrary, amotivation represents the lack of intentionality and motivation. Extrinsically motivated behaviors are placed between these two extremes and they can be expressed in four regulations depending on the degree of self-determination: External, Introjected, Identified, and Integrated. External regulation represents the less autonomous (or more controlled) type of extrinsic motivation, whereas Integrated regulation is the most self-determined extrinsic motivation. An individual externally regulates exercises due to reward contingencies, coercion, or external pressures, whereas in Introjected regulation, behavior is motivated by an internal obligation and a sense of guilt. On the contrary, in Identified regulation the individual consciously recognizes the worth and value of doing exercise. Finally, Integrated regulation means a full endorsement of the exercise and sport with other individual‘s values and identifications (for example, being consistent with a healthy lifestyle, among others) [18].

In accordance with Ryan and Deci [17], each of these regulatory behaviors has its own specific determinants, qualities and phenomenology, as well as different consequences in terms of well-being and performance. Specifically, the more autonomous the motivation is (i.e., Identified, Integrated, and Intrinsic), the greater wellbeing, healthy development, and effective performance will be. In contrast, those regulations considered more controlled (i.e., Introjected and External) have revealed positive associations with impoverished adjustment (see [15] for a review). Autonomous motivation has also been associated with positive body image and healthy eating habits/behaviors, whereas controlled motivation has been inversely related to these outcomes [19]. In addition, concerning moral attitudes and antisocial behavior, research has identified pervasive positive influences of autonomous motivation on keeping winning in perspective and a prosocial moral attitude. In contrast, positive effects of controlled motivation on acceptance of gamesmanship and cheating have been found [20].

### 1.3. Perfectionism and Self-Determination Continuum

A considerable number of studies have paid attention to the relationship between perfectionism and self-determination types of motivation, most of them conducted in the sport domain. Stoeber et al. [21] performed a review of the literature examining the relationship between autonomous and controlled motivation, and the six regulatory styles (Intrinsic, Integrated, Identified, Introjected, External, and Amotivation). In this review, authors concluded that PC is mainly associated with regulatory styles characterized by lower degrees of self-determination, such as Introjected regulation, External regulation, and Amotivation. By contrast, PS is more closely linked to regulatory styles characterized by higher degrees of self-determination (i.e., Intrinsic, Integrated, and Identified regulation). 

Unfortunately, as Stoeber et al. [21] noticed, most of the studies included in their literature review missed the inclusion of some of the six regulatory styles and/or they only differentiate between autonomous versus controlled motivation. From our knowledge, Stoeber et al. [22] carried out the only study that has analyzed the relationship between perfectionism and the full SDT’s motivational continuum. Specifically, this study was conducted in the work domain, with a sample of 131 British employees. Correlational analyses showed positive and significant associations between PS and Intrinsic, Integrated, Identified, and Introjected regulation, as well as negative and significant associations between PS and Amotivation, whereas Non-significant correlations were observed between PS and External regulation. In contrast, positive and significant correlations were obtained between PC and Introjected and External regulations and Amotivation, whereas non-significant correlations were observed between PC and the highest degrees of self-determination (i.e., Intrinsic, Integrated, and Identified). However, the question about how the combination of PS and PC, resulting in different profiles, can lead to different outcomes in terms of the six regulatory styles is still pending analysis.

### 1.4. Perfectionism and Latent Profile Analysis

Although research about perfectionism has been traditionally based on a variable-centered approach, studies from a person-centered approach have increased during the last years. This is because a person-centered approach brings us closer to the “real person” identifying profiles of different levels on perfectionist dimensions, which are, in turn, associated with different outcomes [23]. Of the different person-centered approach techniques, Latent Profile Analysis (LPA) is now considered the most appropriate method as it offers many advantages over traditional ones [24]. Based on this technique, Gilman et al. [25] identified three profiles of perfectionism in a sample of 718 high school students from USA, labeled as *Adaptive Perfectionists* (high PS and low PC), *Maladaptive Perfectionists* (high PS and PC), and *Non-Perfectionists* (comparatively low PS). These three profiles classified, respectively, the 25%, 17%, and 58% of participants. Similar results were found by Moate et al. [26], in a sample of 78 counselor educators from the USA, who identified the following classes: *Adaptive Perfectionists* (61.8%), with high PS and low PC; *Maladaptive Perfectionists* (15.7%), with high PS and PC; and *Non-Perfectionists* (22.25%) with low PS and medium PC. On the other hand, in a sample of 183 undergraduates from Russia, Wang et al. [27] identified *Adaptive Perfectionists* (high PS and relatively low PC), *Maladaptive Perfectionists* (high PS and PC), and *Non-Perfectionists* (low PS and PC), the prevalence of these profiles being 39%, 34%, and 27%, respectively. Lastly, Moate et al. [28], using a sample of 528 doctoral students from the USA, obtained a three class solution: *Adaptive Perfectionists* (high PS and low PC), *Maladaptive Perfectionists* (high PS and low PC), and *Non-Perfectionists* (low PS and moderate PC), representing 58.1%, 28.8%, and 13.1% of the population, respectively. In all the studies cited above, the Revised Almost Perfect Scale (APS-R) [29] was employed to assess perfectionism. 

Using the Sport-Multidimensional Perfectionism Scale-2 (Sport-MPS-2) [30], Pacewicz et al. [31] also identified three profiles of sport perfectionism in North American high-performance athletes. *Pure Personal Standards Perfectionists* (52.6%) obtained high PS and low PC and *Mixed Perfectionists* (27.17%) reported high PS and PC, whereas *Non-Perfectionists* (16.19%) manifested low PS and medium PC.

By contrast, Herman et al. [32], in a sample of African-American sixth grade students, obtained four classes, i.e., *Non-critical or Adaptive,* with high PS and low PC; *Critical or Maladaptive,* with high PS and PC; *Non-Perfectionist* with low PS and PC; and *Non-striving* with severely low scores on PS and low PC. Perfectionism was assessed by using a three-dimensional version of the Child and Adolescent Perfectionism Scale (CAPS-14) [33]. Prevalence for the four classes was 27%, 41%, 23%, and 9%, respectively. 

### 1.5. This Study

This study explored, from a person-centered approach, whether different profiles of perfectionism were differentially associated with the full SDT’s motivational continuum in the specific context of exercise. Specifically, the perfectionism dimensions PC and PS were used as measures to perform a Latent Profile Analysis (LPA) of the data. LPA has received increasing attention as a method of stablishing perfectionism profiles, as has been explained before. Although the class-solution differed from one study to another probably because of the different samples, domains, and measures of perfectionism employed, most of previous research has supported a three-class solution: *Adaptive Perfectionists* (i.e., high PS and low PC), *Maladaptive Perfectionists* (i.e., high PS and PC), and *Non-Perfectionists* (i.e., low PS and PC) [25,26,27,28,31]. In accordance with previous literature [21], if this three-class model fits the data, we hypothesize that: (a) *Adaptive Perfectionists* would experience the highest levels of autonomous motivation (i.e., Intrinsic, Integrated, and Identified); (b) *Maladaptive Perfectionists* would show more controlled motivation (i.e. Introjected, External, and Amotivation), and (c) *Non-Perfectionists* would report the lowest levels of both autonomous and controlled motivation. 

## 2. Materials and Methods 

### 2.1. Participants and Procedures

A total sample of 597 Ecuadorian undergraduates enrolled in a sport science degree program took part in this study (*M*_age_ = 22.08, *SD* = 3.33). Among them, 131 were female (21.94%) and 466 (78.06%) were male. Following the ethical standards established in the 1964 Declaration of Helsinki and its later amendments, written inform consent was requested. The assessment instruments were completed by the participants voluntarily and anonymously in approximately 30 min. A duly trained research team member was always present to explain the procedure to the participants, as well as to solve any questions that may arise.

### 2.2. Instruments

#### 2.2.1. Perfectionism

To measure the two forms of perfectionism (i.e., PS and PC), a multi-measure approach was followed. The Frost’s Multidimensional Perfectionism Scale (FMPS) [34] and the Hewitt’s Multidimensional Perfectionism Scale (HMPS) [35] were employed. Both FMPS and HMPS were adapted into Ecuadorian Spanish using a direct and back-translation method. The FMPS is a 36-item self-report measure that assesses five perfectionism dimensions in a Likert-type format with a five-point response: Concern over Mistakes (CM), Personal Standards (PS), Parental Expectations (PE), Parental Criticism (PC), Doubts about Actions (DA), and Organization (O). The HMPS is a 45-item measure of Self-Oriented Perfectionism (SOP), Socially Prescribed Perfectionism (SPP), and Other-Oriented Perfectionism (OOP) by using a seven-point rating scale response. In accordance with previous research [36], the CM, PE, PC, DA, and SPP are usually employed as indicators of PC, whereas PS, O, SOP, and OOP are commonly used as indicators of PS. An Exploratory Factor Analysis (EFA) with the data at hand supported the structure proposed by Bieling et al. [36], as all dimensions obtained factor loadings above 0.50 in one of the two higher order perfectionism dimensions, with the exception of OOP, whose factor loadings were -0.29 for PS and 0.05 for PC. Therefore, OOP was excluded from the following analysis. The reliability coefficients, Cronbach’s alpha, for the present study were acceptable: CM (α = 0.88), PS (α = 0.88), PE (α = 0.70), PC (α = 0.72), DA (α = 0.71), O (α = 0.85), SOP (α = 0.85), SPP (α = 0.85). 

#### 2.2.2. Self-Determination Continuum

The Behavioral Regulation in Exercise Questionnaire (BREQ-3) validated in a Spanish sample of exercisers [37] was employed. This 23-item instrument assesses the six regulatory styles of the SDT’s motivational continuum in the context of sport and exercise: Intrinsic, Integrated, Identified, Introjected, External, and Amotivation. Responses are scored using a five-point Likert scale. Two Ecuadorian experts verified the adequacy of the items wording to Ecuadorian Spanish. No changes were recommended. The reliability coefficients, Cronbach’s alpha, for the present study were acceptable: Intrinsic (α = 0.78), Integrated (α = 0.78), Identified (α = 0.70), Introjected (α = 0.76), External (α = 0.87), and Amotivation (α = 0.73). 

### 2.3. Data Analysis

Statistics, including Means (*M*), Standard Deviations (*SD*), and Pearson’s correlation coefficients, were calculated for the relationship between PS, PC, and the six factors of the BREQ-3. Effect sizes of these correlations were considered small when values oscillated between 0.10 and 0.30; moderate between 0.30 and 0.50, and large for values ≥0.50 [38].

LPA was conducted to determine whether a latent class structure could be identified on the basis of the two perfectionism dimensions, PS and PC. Statistical analyses begin with a class, which suggests a classification adjustment for all individuals. Next, individuals were successively assigned to an ascending number of classes. The following criteria were considered to define which number of classes best fitted to the data [39]: (a) the lowest values of the Akaike Information Criterion (AIC) and the Bayesian Information Criteria (BIC); (b) *p*-values below 0.05 associated to the Vuong-Lo-Mendell-Rubin Likelihood-Ratio Test (LRT) and the Bootstrap Likeli-hood Ratio Test (BLRT); (c) entropy values close to one. In addition to these indices and statistics, in order to have a meaningful class classification, no solution including small classes was considered (with less than 25 classified cases). 

Subsequently, a multivariate analysis of variance (MANOVA) was performed in order to determine whether there were differences in the mean levels of the six types of motivation regulatory styles (i.e., Intrinsic, Integrated, Identified, Introjected, External, and Amotivation) across the different latent classes, using eta square to determine the magnitude of effect. *Post hoc* tests using the Bonferroni method were conducted to identify between which profiles there were significant differences. In order to calculate the magnitude of these differences, the Cohen’s *d* index was used. This index was interpreted considering Cohen’s criteria [38]: small (*d* = 0.20–-.49), moderate (*d* = 0.50–0.79), and large effect size (*d* ≥ 0.80). 

MPlus Version 8.4 (Muthén & Muthén, Los Angeles, USA) and SPSS 21 (IBM Corporation, Armonk, NY, USA) were used to perform these statistical analyses. 

## 3. Results

### 3.1. Descriptive Statistics and Bivariate Correlations

Descriptive statistics and bivariate correlations among study scales are reported in Table 1. Positive and significant correlations of a moderate magnitude were found between PS and PC. PS also positively and significantly correlated, with small effect sizes, with all SDT’s continuum regulatory styles, with the exception of Amotivation, whose correlations did not reach statistical significance. Similarly, PC significantly and negatively correlated with moderate effect sizes, with Introjected, External, and Amotivation, whereas negative and significant correlations of a small magnitude were found between PC and Intrinsic. 

### 3.2. Perfectionism Profiles

The model fit of the six estimated latent profile solutions are displayed in Table 2. The AIC and BIC had lower values for each class solution that increased one class. A five-class solution obtained the lowest BIC whereas the six-class solution obtained the lowest AIC. However, these two class solutions also presented one class that showed less than 25 participants and were therefore rejected by the established criterion. Regarding LRT, all class solutions, with the exception of the six-class model, presented a *p*-value below 0.05. 

Combining all the criteria, the four-class solution was the most optimal. This model also obtained the highest Entropy value, indicating a good precision in the classification of 80% of cases as estimated by posterior probabilities. 

**Figure 1:**
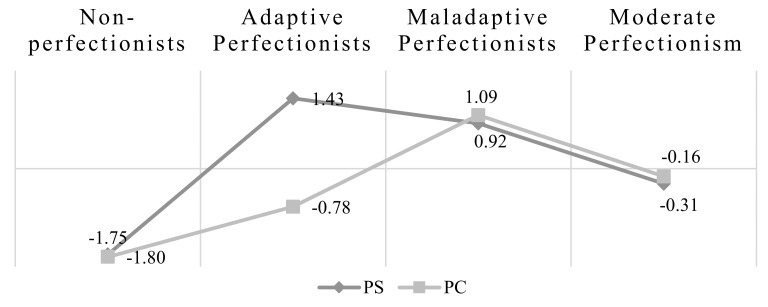
Graphic representation of the standardized average scores for the model of the latent classes.

In terms of interpretability, this four-class solution distinguished the following perfectionism profiles: Class 1 (*N* = 33) reported low levels on both PS and PC dimensions, thus, it was labeled as *Non-Perfectionists*. Class 2 (*N* = 29) was labeled as *Adaptive Perfectionists,* as they reported high PS scores and low PC scores. Class 3 (*N* = 129) was labeled *Maladaptive Perfectionism,* as it classified participants who showed both high PS and PC. Finally, because individuals (*N* = 406) were characterized by medium levels of PS and PC, Class 4 was labeled as *Moderate Perfectionists*. Figure 1 illustrates the standardized means of the two-specific indicators (PS and PC) for each of the four latent classes.

### 3.3. Inter-Profiles Differences in SDT’s Continuum

A MANOVA determined whether these four classes differed in their levels of the six regulatory motivational styles. Statistically significant differences were found for all the variables assessed (Lambda de Wilks = 0.83, *F*_(18,591)_ = 6.32, *p* < 0.001, η^2^ = 0.06). *Non-Perfectionists* obtained the lowest mean scores on Integrated, Identified, Introjected, External and Amotivation, whereas *Moderate Perfectionists* scored the lowest on Intrinsic and Integrated. In contrast, *Adaptive Perfectionists* reported the highest mean scores on Intrinsic, Integrated, and Identified, whereas *Maladaptive Perfectionists* scored the highest on Introjected, External, and Amotivation (see Table 3). 

When examining *post hoc* comparisons (see Table 4), *Maladaptive Perfectionists* reported significantly higher levels of Introjected, External, and Amotivation than the other three profiles of perfectionism. *Moderate Perfectionists* also reported higher scores than *Non-Perfectionists* on Introjected. The magnitudes of these differences were moderate and large for all cases (*d* = 0.55–1.37), with the exception of the *Maladaptive* and *Moderate Perfectionists* contrasts in Amotivation, whose effect sizes where of a small magnitude (*d* = 40). On the other hand, *Adaptive Perfectionists* had significantly higher scores on Intrinsic, Integrated, and Identified when compared with *Moderate Perfectionists*, as well as on Integrated when compared with *Non-Perfectionists*. Moderate and small effect sizes were found for these differences (*d* = 0.47–0.69). 

There were no statistically significant inter-classes differences for the rest of the contrasts analyzed.

## 4. Discussion

The purpose of this study was (a) to examine whether discernible profiles could be identified among undergraduate students enrolled in a sport science degree on levels of PS and PC, and (b) whether these profiles differed in terms of the six types of motivation located on the autonomy-control continuum described by Ryan and Deci [13,18,19] in the specific domain of exercise. Following previous research [25,26,27,28,31], it was hypothesized that a three-class model of perfectionism would be found. However, a four-class model (*Adaptive*, *Maladaptive*, *Non-Perfectionists,* and *Moderate*) obtained a better fit and Entropy values than a three-class model. Of the four classes identified in the current study, three matched those described by previous research for a three-class model: *Adaptive Perfectionists* (high PS and low PC), *Maladaptive Perfectionists* (high PS and PC), and *Non-Perfectionists* (low PS and PC). Additionally, a fourth profile, *Moderate Perfectionists,* representing those participants with medium levels of both PS and PC, was identified. In terms of prevalence, this fourth profile reported the highest prevalence (68%) followed by *Maladaptive Perfectionists* (21%), whereas *Adaptive* (6%) and *Non-Perfectionists* (5%) represented a lower amount of participants. The considerable proportion of individuals classified in *Moderate Perfectionists* in comparison with the other three profiles could be due to the fact that this class represents more normal levels of perfectionism, whereas the other three classify more extreme cases. 

It is not possible to compare the prevalence obtained by *Moderate Perfectionists* with previous research because this class was not previously identified by any LPA [25,26,27,28,31,32]. Regarding the other three classes, as can be deduced from the literature review, there is no consistent results about the prevalence of each class. Thus, although *Adaptive Perfectionists* and *Non-perfectionists* were, respectively, the most and least prevalent classes in most of the studies [27,28,31], *Maladaptive Perfectionists* classified the lowest number of participants in other works [25,26], whereas *Non-Perfectionists* obtained the highest prevalence in the analysis performed by Gilman et al. [25]. Our results are in line with the majority of studies, which found that *Non-Perfectionists* classified the lowest proportion of participants [27,28,31]. 

Overall, differences found between our findings and results from previous research that have examined perfectionism profiles by using LPA [25,26,27,28,31,32] could be due to the different instruments employed to assess perfectionism as well as the sample characteristics. 

In respect of inter-profile differences in the six regulatory styles of motivation, as hypothesized, *Adaptive Perfectionists* experienced the highest levels of Autonomous motivation (i.e., Intrinsic, Integrated, and Identified). However, these differences were only statistically significant in the case of *Adaptive* and *Moderate Perfectionists* contrasts for the three most autonomous regulatory styles, and in the case of *Adaptive* and *Non-Perfectionists* contrasts, for the Integrated style. On the contrary, non-significant differences were found between *Adaptive* and *Maladaptive Perfectionists* profiles in terms of Intrinsic, Integrated, and Identified motivation. Individuals with high levels of PS are characterized by being very organized, persistent, and focused on achieving high standards of performance. Considering that both *Adaptive* and *Maladaptive Perfectionists* profiles are characterized by showing high levels of PS, this fact supports the idea that this perfectionist facet might predispose to more autonomous motivated regulations for practicing physical activity, in accordance with previous research [21]. However, *Adaptive Perfectionists* (high PS and low PC), when compared with *Maladaptive Perfectionists* (high PS and PC), would be more likely to be motivated for exercise with a sense of personal control over behavior, including enjoyment and personal affinity to sport and exercise [40]. That is, *Adaptive Perfectionists* could benefit more from their PS’s motivational outcomes than *Maladaptive Perfectionists* because of the negative motivational consequences of having high PC. In fact, as expected, *Maladaptive Perfectionists* experienced the highest levels of Amotivation, External, and Introjected motivation when compared with the other three profiles. It is important to mention that the majority of these differences involved moderate and even large effect sizes, indicating that they are not only significant, but they also represent theoretical relevance and practical consequences for daily life [41].

These results could be due to the fact that *Maladaptive Perfectionists* was the only perfectionist class characterized by high PC, and they would be in line with previous studies that positively associate PC with regulatory styles characterized by lower degrees of self-determination [21]. These results evidence the fact that *Maladaptive Perfectionists* present maladjusted motivational trends. On the one hand, *Maladaptive Perfectionists* are more likely to experience motivations for doing exercise only partially internalized into the self (i.e., Introjected and External). This might be explained because PC is characterized by harsh self-criticism, fear to failure, and the belief that the environment is highly demanding and critical. Hence, because in Introjected and External regulations the individual is motivated, respectively, by internal and external rewards or punishments [17], we speculate that *Maladaptive Perfectionists* would be more introjected and externally regulated by a sense of coercion, internal contingencies, and external pressures [40] when they expected success. On the other hand, after repeated failures or when a certain failure is expected, *Maladaptive Perfectionists* could experience Amotivation, that is, a lack of intention and commitment [17] to exercise, in order to avoid a certain punishment or because they feel they are not able to effectively attain the expected outcomes. 

Additionally, it was expected that *Non-Perfectionists* would report the lowest levels of both autonomous and controlled motivation. Nevertheless, this hypothesis was partially supported by the results of this study. Thus, *Non-perfectionists* reported the lowest levels of Integrated, Introjected, External, and Amotivation. However, when inter-profile contrasts were analyzed, statistically differences only emerged for *Non-Perfectionists* and *Adaptive Perfectionists* comparisons on Integrated motivation, *Non-Perfectionists* and *Maladaptive Perfectionists* comparisons on Introjected, External, and Amotivation, and between *Non-Perfectionists* and *Moderate Perfectionists* on Introjected regulation. Hence, *Non-Perfectionists* individuals would be characterized by having what Vansteenkiste et al. [42] called a “low quantity motivation” profile. Although, apparently, these *Non-perfectionists* outcomes could be interpreted as the most negative on the basis of quantitative theories of motivation, research has evidenced that the quality of motivation matters [42]. In fact, it seems that “the presence of controlled motivation, next to either a high amount of autonomous motivation or a low amount of autonomous motivation, yields no benefits at all” [42] (p. 684). Controlled motivation (i.e., Introjected and External regulations) has been, indeed, associated with poorer wellbeing, healthy eating habits and behaviors, moral attitudes, experience, and performance outcomes in the context of sport and exercise (e.g., [15,19,20]). Consequently, *Maladaptive Perfectionists* would be more likely to experience a more damaging motivational orientation than *Non-Perfectionists*. 

Several limitations of this study should be mentioned. First of all, having used a cross-sectional design does not allow us establishing causality relationships. Longitudinal and experimental perspectives would help to clarify the relationship (and its direction) between perfectionism and the SDT continuum. Additionally, it is important to underline that our participants were recruited from a sample of undergraduate students enrolled in a sport science degree from Ecuador. Thus, these results should be generalized with caution to other samples such as professional athletes or different ethnic and age groups. Additionally, it would be interesting to analyze whether these same findings can be extrapolated to other life domains, such as academic. Furthermore, women were underrepresented in our study, because only 21.94% of the total sample were female. Future studies might examine gender differences performing separate LCA for males and females as well as testing whether these profiles show similar or different motivational outcomes across sex. In a similar way, future studies might also explore whether these results vary across age and socio-economic status.

## 5. Conclusions

In spite of the limitations, this is, from our knowledge, the first study that has addressed the relationship between perfectionism and motivation using a person-centered approach. Moreover, the fact that both constructs have been examined considering the six regulatory styles of the autonomy-control continuum in the specific domain of exercise is also a novelty. In this research, four types of perfectionism profiles consistently emerged: *Adaptive Perfectionists*, *Maladaptive Perfectionists*, *Moderate Perfectionists*, and *Non-Perfectionists*. In terms of prevalence, a considerable amount of participants classified in these three first profiles in comparison with *Non-Perfectionists* evidence that perfectionism is a widely extended trait of personality. Especially striking is the fact that two out of 10 individuals are at a potential risk of mental health problems because of their high levels of dysfunctional perfectionism. These results are in line with Flett and Hewitt [43], who recently have referred to this widespread and growing prevalence of perfectionism as the “perfectionism pandemic.” Therefore, specific interventions should be addressed to *Maladaptive Perfectionists* for reducing their high levels of perfectionism, especially those more dysfunctional forms, such as PC. Overall, in light of SDT theory [13,17], the results obtained indicate that *Adaptive Perfectionists* would report the most beneficial outcomes in terms of motivation since they tend to exercise as emanating from, and an expression of, one’s self. In contrast, *Maladaptive Perfectionists* would display the poorer motivational functioning, as they tend to perceived external or internal pressures and duties or even a lack of intentionality and motivation to exercise. In accordance with the SDT theory [13,17], contexts can only produce autonomous regulation if they support autonomy, and therefore, allow a person to feel competent, related, and autonomous. In the same way, contexts can produce external regulation if there are relevant threats or rewards and the person feels competent enough to fulfill the demands. Hence, it is recommended that trainers and exercise instructors take special attention to maladaptive perfectionist exercisers, implementing motivational techniques that emphasize intrinsic goals in an autonomy-supportive way [44]. 

## Figures and Tables

**Table 1:** Descriptive statistics and bivariate correlations between Perfectionistic Strivings (PS), Perfectionistic Concerns (PC) and the Behavioral Regulation in Exercise Questionnaire (BREQ-3) subscales.

	PS	PC	*M*	*SD*
PS	---	---	90.70	15.01
PC	0.44 **	---	97.27	17.48
Intrinsic	0.19 **	−0.09 *	13.22	2.81
Integrated	0.25 **	−0.05	12.74	3.02
Identified	0.23 **	−0.06	9.67	2.22
Introjected	0.19 **	0.43 **	5.84	4.15
External	0.12 **	0.40 **	3.39	4.06
Amotivation	0.08	0.35 **	4.20	3.96

* *p* < 0.05; ** *p* < 0.01.

**Table 2:** Fit indexes of the results of the Latent Profile Analysis (LPA).

Account of Classes	*AIC*	*BIC*	*LRT*	*BLRT*	Entropy	Size
2 classes	3320.46	3351.20	0.017	0.00	0.43	0
3 classes	3264.10	3308.01	0.012	0.00	0.73	0
4 classes	3232.44	3289.53	0.003	0.00	0.80	0
5 classes	3211.09	3281.36	0.033	0.00	0.74	1
6 classes	3199.44	3282.88	0.120	0.00	0.77	1

Akaike Information Criterion (AIC) and the Bayesian Information Criteria (BIC); LRT = Vuong-Lo-Mendell-Rubin Likelihood-Ratio Test; BLRT = Bootstrap Likelihood Ratio Test.

**Table 3:** Means, standard deviations, and inter-class statistic signification on the six regulatory styles.

SDT’s Continuum	*Non-Perfectionists*		*Adaptive Perfectionists*		*Maladaptive Perfectionists*		*Moderate Perfectionists*		Statistical Significance and Effect Sizes		
	*M*	*SD*	*M*	*SD*	*M*	*SD*	*M*	*DT*	*F_(3, 593)_*	*p*	η^2^
Intrinsic	13.69	2.77	14.37	2.67	13.52	2.41	13.01	2.92	3.21	0.023	0.02
Integrated	11.87	3.40	14.06	2.90	13.25	2.62	12.56	3.08	4.55	0.004	0.02
Identified	9.72	2.37	11.00	1.71	9.99	1.94	9.47	2.29	5.52	0.001	0.03
Introjected	2.54	3.01	3.82	4.48	8.04	4.23	5.56	3.85	24.48	<0.001	0.11
External	1.39	2.70	1.89	3.42	5.25	4.98	3.06	3.66	14.81	<.0001	0.07
Amotivation	2.42	3.34	3.00	4.55	5.55	4.65	4.01	3.61	8.68	<0.001	0.04

SDT = Self-Determination Theory.

**Table 4:** Cohen’s d indexes for post-hoc contrasts between the mean scores obtained by the four classes on the six motivational regulatory styles.

SDT’s continuum	*Non-Perfectionists vs.* *Adaptive P.*	*Non-Perfectionists vs. * *Maladaptive P.*	*Non-Perfectionists vs. * *Moderate P.*	*Adaptive P vs. * *Maladaptive P.*	*Adaptive Perfectionists vs. * *Moderate P.*	*Maladaptive Perfectionists vs. * *Moderate P.*
Intrinsic	---	---	---	---	0.47 *	---
Integrated	0.69 *	---	---	---	0.49 *	---
Identified	---	---	---	---	0.68 **	---
Introjected	---	1.37 ***	0.80 ***	0.79 ***	---	0.63 ***
External	---	0.84 ***	---	0.71 **	---	0.55 ***
Amotivation	---	0.71 **	---	0.55 *	---	0.40 **

SDT = Self-Determination Theory; P. = Perfectionists; * *p* < 0.05; ** *p* < 0.01; *** *p* < 0.001.
